# Unveiling the Skin Anti-Aging Potential of the Novel *Spirulina platensis* Extract Elixspir^®^

**DOI:** 10.3390/ijms262311372

**Published:** 2025-11-25

**Authors:** Chiara Donati, Giulia Nerina Nardone, Vera Mason, Emanuela Di Gregorio, Irene Ragusa, Emanuele Amadio, Eleonora Zampieri, Rebecca Bassetto, Valentina Gandin, Samuele Zanatta

**Affiliations:** 1 Dipartimento di Scienze del Farmaco, Via F. Marzolo 5, 35131 Padova, Italy; chiara.donati.1@phd.unipd.it (C.D.); eleonora.zampieri.1@unipd.it (E.Z.); 2 Labomar S.p.a., Via F. Filzi 33, 31036 Treviso, Italy; giulianerina.nardone@labomar.com (G.N.N.); vera.mason@labomar.com (V.M.); emanuela.digregorio@labomar.com (E.D.G.); irene.ragusa@labomar.com (I.R.); emanuele.amadio@labomar.com (E.A.); rebecca.bassetto@labomar.com (R.B.)

**Keywords:** spirulina aqueous extract, skin anti-aging, antioxidant effect, photoprotection

## Abstract

*Arthrospira platensis* (commonly known as Spirulina platensis) is a blue-green microalga increasingly used in skincare due to its antioxidant and dermo-protective properties, primarily attributed to components such as phycocyanin and carotenoids. However, the intense blue color of phycocyanin can limit its cosmetic appeal. In this study, we investigated the antioxidant, anti-inflammatory, skin lightening and photoprotective activity of Elixspir^®^, a novel light-colored aqueous extract of Spirulina, using both 2D and 3D skin cell models. We demonstrated that Elixspir^®^ exerts strong antioxidant and cytoprotective effects by reducing intracellular ROS levels and modulating cellular thiol redox state. Its anti-pigmentation potential was supported by tyrosinase inhibition, while anti-inflammatory activity was principally due to ability to reduce PGE2 levels. Finally, we demonstrated an unprecedented photoprotective effect of Elixspir^®^, highlighting its potential as a novel active ingredient for skin defense against environmental stressors. Overall, these results provide a molecular-level understanding of Elixspir^®^ multifunctional bioactivity and support its application as a skin-lightening, anti-inflammatory, antioxidant, and photoprotective ingredient in the formulation of innovative skin anti-aging treatments.

## 1. Introduction

Aging is a complex biological process characterized by a gradual decline of physiological functions, affecting different tissues and organs, and, in particular, the skin [1,2]. The skin is the largest body organ, mainly composed of a network of components, like collagen, elastin, glycoproteins, and hyaluronic acid. With aging, the level of these components progressively decreases, leading to a loss of skin strength and flexibility, which results in visible wrinkles, fine lines, sagging, and uneven skin tone. These changes are a result of a combination of intrinsic and extrinsic factors [3,4,5,6,7].

Intrinsic factors, such as genetic predispositions and hormonal fluctuations, play an important role in aging [7,8].

**Figure 1:**
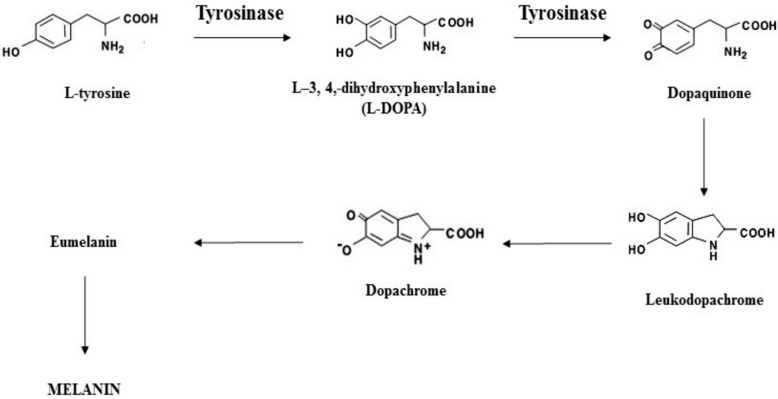
Melanogenesis pathways and tyrosinase involvement.

Extrinsic factors, on the other hand, include all those external elements that accelerate aging processes. Among them, environmental stressors like pollution can generate free radicals, leading to oxidative stress and inflammation, thus further accelerating aging-related skin damage. However, ultraviolet (UV) radiation from the sun represents the most relevant extrinsic factor to skin aging, also known as photoaging. UV exposure has been demonstrated to strongly induce oxidative processes, ultimately resulting in the generation of reactive oxygen species (ROS), leading to premature aging [9,10]. Such cellular ROS overproduction can trigger several reactions in the body, like the consumption of antioxidant defenses and the increased expression of specific enzymes such as matrix metalloproteinase 1 (MMP-1) and tyrosinase [11,12]. Tyrosinase plays a key rate-limiting role in the biosynthesis of melanin [13,14,15], a natural pigment responsible for skin color, which provides protection against the deleterious effects of UV irradiation [16,17] (Figure 1). Despite its usefulness, the accumulation of an abnormal amount of melanin in several parts of the skin results in the development of pigmented patches that might cause social discomfort and, in some cases, can also be a sign of other medical conditions [16,17,18,19].

The beauty and skincare industry has been actively searching for effective ingredients capable of slowing down and reducing the visible signs of skin aging, particularly those caused by extrinsic factors, in order to develop more targeted and preventative anti-aging skin solutions [8,19].

To counter ROS molecule action, several antioxidants (such as resveratrol and vitamin C) are included in formulations [20], and in those addressed to skin-brightening, they are often associated with niacinamide, alpha arbutin, resveratrol, and licorice extract, which work by inhibiting melanin production, promoting skin renewal, and reducing oxidative stress [21,22,23]. Among the ingredients used in depigmentation preparations, kojic acid is widely used due to its ability to target different stages of melanin production: it acts as a chelator of divalent ions, as a free radical trapper, and as a tyrosinase inhibitor as well. However, its cytotoxic effect and instability over time have led the Scientific Committee on Consumer Products (SCCP) to limit its use by reducing the maximum acceptable concentration to less than 1% [15]. Moreover, the Japanese officials also sought to ban the use of kojic acid in cosmetic products due to its carcinogenicity [24].

Despite this ban, kojic acid and other synthetic molecules are often still introduced into cosmetic formulations. In contrast, botanicals and natural ingredients represent safer alternatives, as they may possess a better biocompatibility profile as compared with synthetic compounds. This drives research efforts towards the development of effective natural skin-whitening ingredients.

An intriguing active component recently explored in numerous anti-aging experimental formulations is *Arthrospira platensis*, commonly known as *Spirulina platensis*. Spirulina is a type of blue-green algae rich in nutrients like vitamins, minerals, and other antioxidants [25], which can effectively counteract the aging process. Moreover, its extract has gained attention in skincare for its potential to protect against environmental damage [26]. Notably, the two main compounds present in several spirulina extracts are phycocyanin and carotenoids, which contribute to its antioxidant and anti-inflammatory properties, effectively neutralizing free radicals and mitigating inflammation associated with skin aging [27]. Phycocyanin is one of the major pigments in spirulina, responsible for its distinctive blue-green color. This compound has exhibited strong antioxidant activity, effectively scavenges free radicals, and protects cells from oxidative stress [28]. Similarly, spirulina carotenoids, including beta-carotene and zeaxanthin, are known for their antioxidant and photoprotective properties. Furthermore, spirulina is a natural source of superoxide dismutase (SOD), an enzyme that catalyzes the breakdown of superoxide radicals [29,30].

In addition to the antioxidant potential, spirulina is also known to exert a variety of immunomodulatory and anti-inflammatory activities by regulating key cytokines. Mechanistic studies performed both in cell and rat models showed that its antioxidant, immunomodulatory, and anti-inflammatory activities are consistent with the ability to regulate the ERK1/2, JNK, p38, and IκB signaling pathways [31,32].

Strongly inspired by the previously reported studies on Spirulina, we developed a novel Spirulina aqueous extract, Elixspir^®^, characterized by low levels of blue phycocyanins. This characteristic offers a relevant advantage to cosmetic formulators; indeed, the unique light-colored extract solution overcomes the limits of traditional enriched phycocyanin spirulina extracts, whose blue color can strongly restrict their use in cosmetic products. In the present research study, the biocompatibility, antioxidant, and anti-inflammatory activity of Elixspir^®^ were validated on suitably developed 2D and 3D cellular models. Moreover, we clearly demonstrated Elixspir^®^’s potential as a skin-lightening and photoprotective ingredient. In more detail, we evaluated the ability of Elixspir^®^ to directly inhibit tyrosinase enzymes and to decrease melanin levels in a human reconstructed skin model. Lastly, we provided preliminary evidence of the photoprotective effect of Elixspir^®^, thus underlining future prospective applications of Spirulina extract in the development of sun-protective products.

## 2. Results

This study investigates the potential of Elixspir^®^ as a well-aging active ingredient for topical formulations, with a focus on its antioxidant, anti-inflammatory, and brightening properties. Initial investigations involve assessing Elixspir toxicity through comprehensive evaluations of biocompatibility and phototoxicity/photoprotection.

### 2.1. Evaluation of Elixspir^®^ Safety

The biocompatibility profile of Elixspir^®^ in V-79, HEK-293, HFF-1, and HaCaT cells was determined using the MTT assay. Biocompatibility assays demonstrated that Elixspir, even at relatively high concentrations, did not significantly compromise cell viability in both 2D and 3D models, thus attesting to its safety and indicating its potential applicability for topical uses (Table 1 and Table 2).

**Figure 2:**
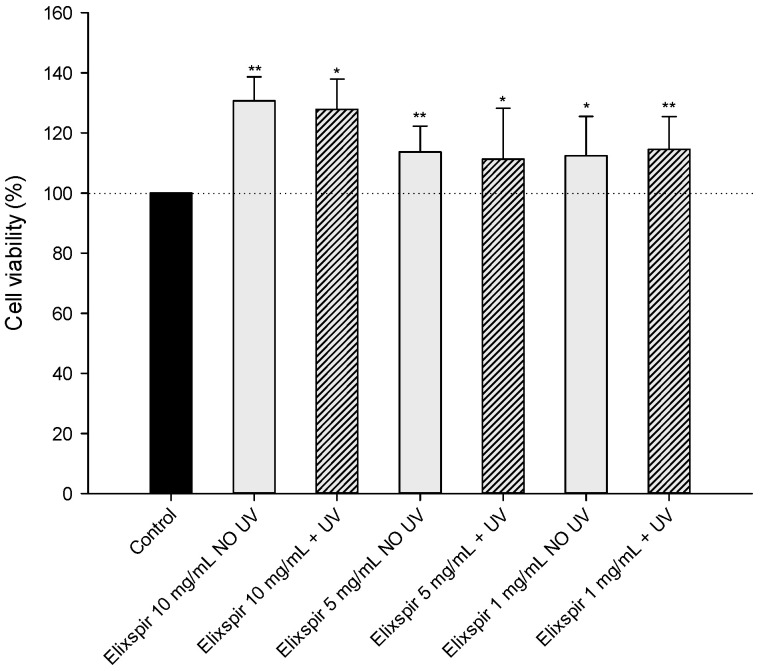
Evaluation of the phototoxicity of different concentrations of Elixspir^®^ in the HEK-293 cell line. Cells were treated for 24 h with irradiated/non-irradiated samples of Elixspir^®^. Cell viability was estimated by means of the MTT test. Error bars indicate S.D. * *p* < 0.05, ** *p* < 0.01 compared with control.

In order to evaluate the potential applicability of Elixspir^®^ in cosmeceutical formulations, the phototoxicity of Elixspir^®^ was initially assessed by irradiating various concentrations of the extract, which were subsequently tested on the HEK-293 cell line. As shown in Figure 2, the different irradiated sample solutions did not exhibit phototoxicity towards the HEK-293 cell line. In fact, cell viability percentage was comparable or, in the case of Elixspir^®^ at 1 mg/mL, even higher than that of the non-irradiated control and of the corresponding non-irradiated solutions. It is important to highlight that treatment with Elixspir^®^ at all tested concentrations, particularly at 10 mg/mL, significantly increases cellular viability.

To further validate Elixspir^®^’s safety profile, we also assessed skin irritation potency. The latter is one of the three toxicological endpoints to be always addressed in a biological risk assessment, whatever the category of the device/product. The Elixspir^®^ skin-irritation profile was evaluated by using the SkinEthic™ RHE model, an in vitro reconstructed epidermis from human primary keratinocytes. The SkinEthic™ RHE model mimics human epidermal morphology and physiology and is also validated as a full in vitro replacement method for animal testing to assess the skin corrosion and the skin irritation potential of chemicals [33,34].

**Figure 3:**
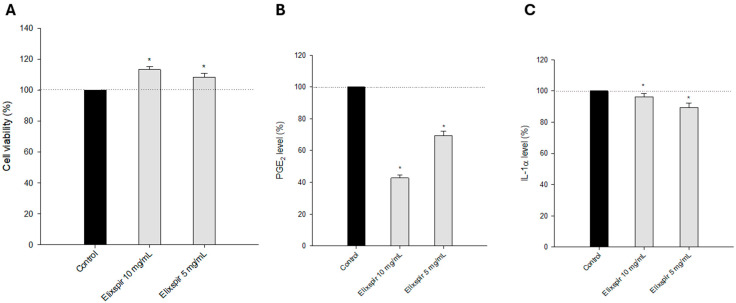
Evaluation of the skin irritation potential of Elixspir^®^ in the SkinEthic™ RHE model. Tissues were incubated at 37 °C with Elixspir for 42 min at room temperature. After exposure, the RHE tissues were rinsed and then further incubated for 42 h at 37 °C. Tissue viability (**A**) was assessed by the MTT reduction assay. PGE2 (**B**) and IL-1α (**C**) were evaluated in tissue lysate by specific ELISA assays following the manufacturer’s instructions. Error bars indicate S.D. * *p* < 0.05 compared with control.

As can be clearly seen in Figure 3A, treatment with Elixspir^®^ solutions at two different concentrations did not induce any reduction in cell viability and, on the contrary, slightly increased the percentage of cell viability in a dose-dependent manner compared to the untreated control.

In addition, quantification of PGE2 and IL-1α release in the tissue medium was also assessed as a potential complementary endpoint to cell viability for classifying Elixspir extract. In accordance with cell viability detection performed in the SkinEthic™ RHE model, Elixspir^®^ did not induce any increase in inflammation mediators, such as PGE2 (Figure 3B) or IL-1α (Figure 3C).

### 2.2. Evaluation of Elixspir Antioxidant, Anti-Inflammatory, Anti-Tyrosinase and Photoprotective Properties

After confirming the safety profile of Elixspir^®^, a comprehensive assessment of its properties, including antioxidant, anti-inflammatory, anti-tyrosinase, and photoprotective effects, was performed.

The ability of Elixspir^®^ to neutralize free radicals and mitigate oxidative stress in cells was shown by means of four different methodological approaches.

**Figure 4:**
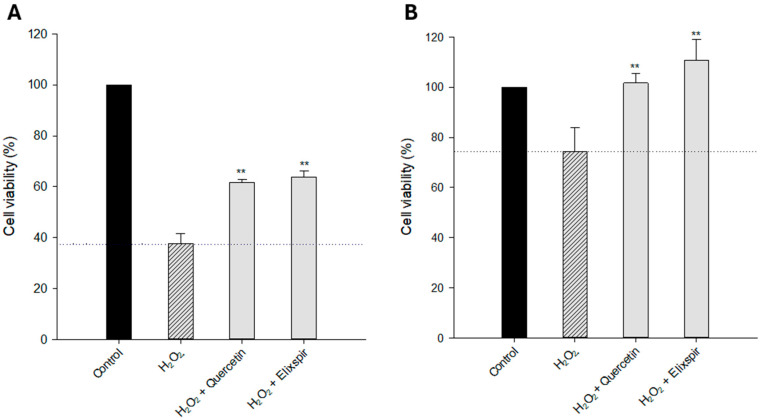
Cytoprotective effect against oxidative damage induced in the human HEK-293 cell line in 2D (**A**) and 3D (**B**) systems. Cells were pretreated with Elixspir^®^ or quercetin and subsequently exposed to H_2_O_2_. Cell viability was estimated by means of the MTT (**A**) or APH (**B**) test. Error bars indicate S.D. ** *p* < 0.05 compared to treatment with H_2_O_2_.

Firstly, the cytoprotective effect against oxidative stress was evaluated on 2D and 3D cell models. Cells were pretreated with Elixspir^®^ or quercetin, a well-known antioxidant and cytoprotective compound, and the oxidative damage was induced by H_2_O_2_ addition. As shown in Figure 4, the percentage of viability of HEK-293 cells was significantly reduced by treatment with H_2_O_2_ (residual viability of 38% and 74% compared to untreated cells in 2D and 3D, respectively). Remarkably, Elixspir^®^ showed a statistically significant cytoprotective effect against the oxidative stress induced by treatment with H_2_O_2,_ which was much more evident in 3D cell cultures than in 2D ones. Actually, in 3D systems, pretreatment with Elixspir^®^ resulted in a cytoprotective effect even higher than the reference antioxidant compound quercetin.

These data clearly showed that Elixspir^®^ exhibits a prominent cytoprotective effect against oxidative damage. To shed light on the mechanism accounting for this activity, the ability of Elixspir^®^ to neutralize free radicals was initially assessed by the DPPH scavenging assays. This method involves measuring the ability of Elixspir^®^ to neutralize DPPH radicals, providing quantitative data on its direct free radical-scavenging capacity. In the test, a DPPH solution is mixed with the test compound, and the change in absorbance at 517 nm is measured over time. The decrease in absorbance is proportional to the scavenging of the DPPH radical by the test compound and is used to calculate the antioxidant activity [35,36].

Remarkably, Elixspir^®^ showed an IC_50_ of 39 µg/mL, clearly attesting to its potential as a prominent antioxidant. However, its potency was relatively lower compared to the reference antioxidant compound vitamin C, which exhibited an IC_50_ of 1 µg/mL.

Subsequently, the ability of Elixspir^®^ to counteract cellular production of ROS after induction of a pro-oxidant stimulus with H_2_O_2_ was also assessed in human HEK-293 cells.

As shown in Figure 4A, pretreatment of HEK-293 cells with Elixspir^®^ significantly reduces the cellular basal production of ROS induced by oxidative stress. Specifically, its ability to neutralize ROS production was even higher than that induced by the reference antioxidant compound, quercetin.

As it is well known that thiols play an essential role in redox reactions within cells and act as antioxidants by scavenging ROS and protecting cells from oxidative damage, we have also assessed the effect induced by Elixspir^®^ in terms of modulation of thiol redox state by using the Ellman assay.

**Figure 5:**
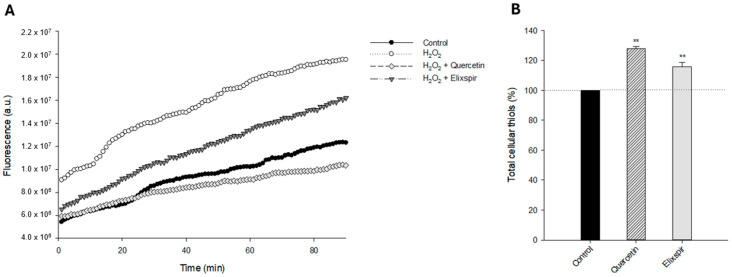
Antioxidant mechanism. (**A**) Evaluation of ROS production. HEK-293 cells were preincubated with Elixspir^®^ or quercetin for 4 h and subsequently were exposed to H_2_O_2_ for 2 h. Afterwards, cells were washed and incubated with PBS/10 mM glucose medium for 20 min at 37 °C in the presence of 10 μM CM H_2_DCFDA. (**B**) Evaluation of cellular thiol redox status. HEK-293 cells were incubated for 24 h with Elixspir^®^ or quercetin. The sulfhydryl group amount was determined by the DTNB assay. Error bars indicate S.D. ** *p* < 0.05 compared with control.

As shown in Figure 5B, HEK-293 cells treated with Elixspir^®^ or quercetin showed a significantly higher level of reduced thiols compared to control cells. In particular, the novel Spirulina extract was able to induce a 15% increase in cellular sulfhydryl content. These results suggest that, in addition to a direct ROS modulation effect, the antioxidant action of Elixspir^®^ also involves a thiol-mediated redox mechanism.

Altogether, these results emerging from different methodological approaches allow us to draw a comprehensive mechanistic understanding of Elixspir^®^ antioxidant activity. These findings not only contribute to understanding its antioxidant profile but also provide valuable information for formulating skincare products with potential protective and anti-aging benefits.

Concurrently, potential anti-inflammatory properties have been investigated to understand the effectiveness of Elixspir^®^ in alleviating inflammation, which is a key factor in various skin conditions.

**Figure 6:**
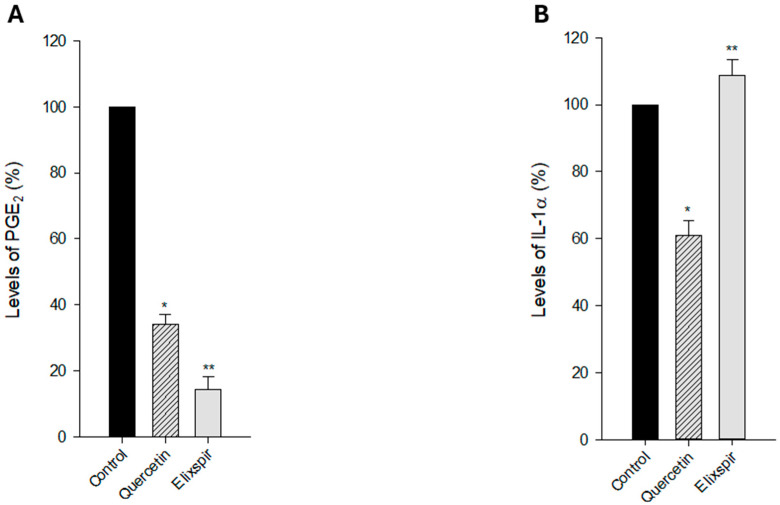
Anti-inflammatory potential. PGE_2_ (**A**) and IL-1α (**B**) levels in HEK-293 cells. Cells were treated with Elixspir^®^ for 24 h, and the quantification of PGE2 and IL-1α was determined by using a competitive immunological ELISA assay, following the manufacturer’s instructions. Error bars indicate S.D. * *p* < 0.1; ** *p* < 0.05 compared with control.

PGE_2_ is considered a pro-inflammatory mediator because it contributes to the characteristic signs of inflammation, including redness, heat, swelling, and pain. Consequently, understanding the role of PGE_2_ in inflammation has important implications for the development of therapies to modulate the inflammatory response in various diseases and conditions [37]. In the same manner as PGE_2_, interleukin-1 alpha (IL-1α) is a pro-inflammatory cytokine that plays a significant role in the initiation and regulation of inflammation. It is produced by various cell types, including immune cells, and is involved in both acute and chronic inflammatory responses [38]. On these bases, we assessed the ability of Elixspir^®^ to modulate PGE_2_ and IL-1α levels in human HEK-293 cells. As shown in Figure 6A, treatment of HEK-293 cells with Elixspir^®^ significantly reduces PGE_2_ levels; in particular, it determined a reduction of 85.6% of PGE_2_ levels compared to control. On the contrary, Figure 6B illustrates that treatment with Elixspir of HEK-293 cells did not result in a decrease in the concentration of IL-1α.

These results are consistent with those obtained in the SkinEthic™ RHE model and suggest that the anti-inflammatory potential of Elixspir^®^ is mainly mediated by an outstanding ability to modulate prostaglandin levels, particularly PGE_2_.

Inhibition of tyrosinase enzymes directly affects melanin production and helps to remove skin hyperpigmentation. Accordingly, extracts that have an inhibitory effect on melanin formation would be a good choice for the cosmetic purpose of whitening the skin. To understand the potential of Elixspir^®^ as a skin-lightening agent, we evaluated its tyrosinase inhibitory effect in comparison to kojic acid, a well-known tyrosinase inhibitor. As shown in Figure 6A, Elixspir^®^ elicited a dose-dependent inhibition of the enzyme. In more detail, Elixspir^®^ was able to inhibit 51% of enzyme activity at the lower tested dose, reaching 100% inhibition with the highest concentration (30% *v*/*v*).

In addition, the ability to modulate melanin production was also assessed in vitro in a more representative environment by employing the RHPE model delivered by SkinEthic.

**Figure 7:**
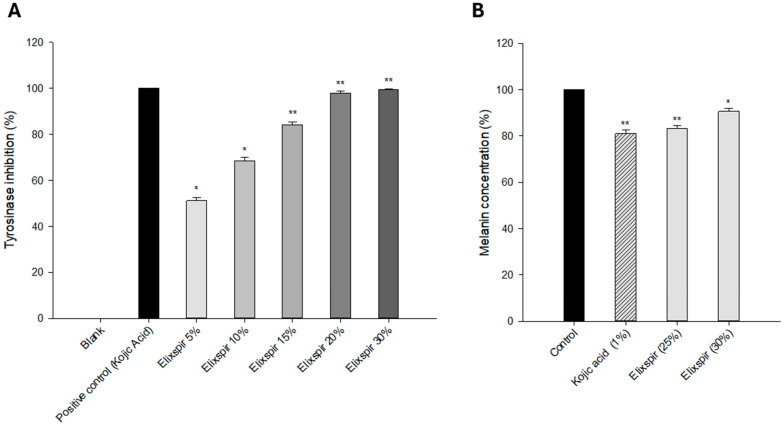
Anti-tyrosinase activity. (**A**) The inhibitory effect of Elixspir^®^ on tyrosinase was evaluated in a cell-free system by means of the DOPA oxidase activity assay as reported in the experimental section. (**B**) Percentage of melanin content in reconstructed human pigmented epidermis after daily application of Elixspir^®^ or kojic acid solution. Error bars indicate S.D. * *p* > 0.1; ** *p* < 0.05 compared with control.

RHPE model tissues were daily treated with Elixspir^®^ (25% *w*/*w* and 30% *w*/*w*) or kojic acid (1% *w*/*w*) solutions for 4 consecutive days and were used as a positive control. As evident in Figure 7B, treatment with Elixspir^®^ solutions, particularly at the 25% concentration, induced a reduction in the amount of tissue melanin, which was comparable to that determined by kojic acid, known for its whitening properties and the ability to inhibit melanin production, used as a positive control.

Overall, these results suggest that Elixspir^®^ could serve as an effective and safer alternative to kojic acid for inhibiting tyrosinase and modulating the melanogenesis process. These findings underscore the potential of Elixspir^®^ as a promising ingredient in formulations targeting skin hyperpigmentation.

### 2.3. Photoprotective Effect

In view of employing Elixpir in topical products, we thought it would be of interest to verify its ability to protect skin cells from phototoxicity. The examination of photoprotective properties aimed to determine its efficacy in shielding the skin against the harmful effects of UV radiation. In fact, photoprotection is vital for preventing sun-induced damage and preserving skin health.

**Figure 8:**
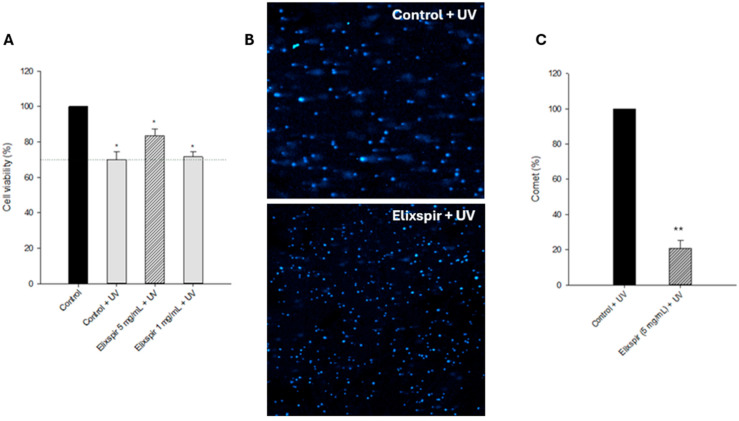
Photoprotective activity. (**A**) Photoprotection of different concentrations of Elixspir^®^ in the HEK-293 cell line. Cells were pre-treated for 24 h with Elixspir^®^ and then irradiated for 30 min at 5 J/cm2 (UVA range). Cell viability was estimated by means of the MTT test. (**B**) Comet assay. HEK-293 cells were pretreated for 6 h with Elixspir^®^ and then irradiated. Representative pictures captured with a Zeiss LSM-800 confocal microscope (left, 10× magnification). (**C**) Relative percentage of comets (number of cells forming a comet/total number of cells) detected in two randomly captured fields from three independent experiments (right). Error bars indicate S.D. * *p* > 0.1; ** *p* < 0.05, compared with control.

HEK-293 cells were pre-treated with Elixspir for 24 h and then irradiated for 30 min at 5 J/cm^2^ (UVA range), considering one microplate without irradiation as a control. Photoprotection was assessed in terms of both cell viability (Figure 8A) and DNA damage (Figure 8B,C).

As illustrated in Figure 8A, pre-treatment with Elixspir^®^ at 5 mg/mL led to a slight increase in cell viability when compared to the irradiated control cells. Based on this result, to further strengthen the hypothesis of its photoprotection activity, we evaluated DNA fragmentation within intact HEK-293 cells exposed to UV irradiation. By employing the alkaline single-cell gel electrophoresis assay (Comet assay, Figure 8B,C) we clearly showed that pretreatment with Elixspir^®^ strongly decreased the electrophoretic migration of the DNA fragments. Figure 8C also depicts the relative percentage of comets and the number of cells forming a comet relative to the total number of detected cells for each condition in two randomly captured fields from two independent experiments.

## 3. Discussion

Research into skin anti-aging agents has become increasingly important due to the growing need to understand and mitigate the biological processes underlying skin aging, with the aim of developing effective ingredients that slow or reverse age-related changes. Among these, botanical and natural ones have emerged as promising alternatives due to their reduced toxicity compared to synthetic compounds and a generally favorable safety profile. This shift has intensified research efforts focused on identifying safe and effective natural ingredients for cosmetic purposes, including skin-whitening and anti-aging applications.

A recently explored and increasingly popular component in anti-aging formulations is Spirulina, a blue-green microalga known for its rich profile of nutrients, including proteins, vitamins, minerals, and antioxidants. Its use in skincare has garnered attention due to its ability to promote collagen synthesis, improve skin elasticity, and offer protection against oxidative and environmental stressors. However, conventional spirulina extracts rich in phycocyanin impart a strong blue hue, limiting their use in visually appealing cosmetic products.

In this study, we investigated Elixspir^®^, a novel light-colored aqueous extract of Spirulina, to evaluate its safety and potential multifunctional bioactivities in the context of well-aging skin formulations. Our research focused on assessing its antioxidant, anti-inflammatory, skin-brightening, and photoprotective properties through a variety of in vitro models and methodological approaches.

Before exploring functional bioactivities, Elixspir^®^’s safety profile was comprehensively established across multiple skin-relevant models. Elixspir^®^ demonstrated excellent biocompatibility in V-79, HEK-293, HFF-1, and HaCaT cells, showing no cytotoxicity even at high concentrations. In addition, the SkinEthic™ RHE and RHPE 3D tissue models confirmed no irritation potential.

As one of the key drivers of skin aging is oxidative stress, which disrupts cellular homeostasis, damages macromolecules, and accelerates dermal degeneration, we tested Elixspir^®^ antioxidant potential by means of selected cell-free and in-cell experiments. Elixspir^®^ exhibited strong antioxidant activity as evidenced by multiple and complementary approaches that provide both phenotypic and molecular-level data. At the molecular level, Elixspir^®^ displayed direct free radical scavenging capacity, eliciting an IC_50_ of 39 µg/mL in the DPPH assay. Even if not as potent as vitamin C (IC_50_~1 µg/mL), this is significantly higher than typical natural extracts, including those derived from green tea or pomegranate [39], suggesting its relevance in antioxidant skincare. Consistently, in cell-based assays, pretreatment with Elixspir^®^ significantly mitigated the cytotoxic effects of H_2_O_2_ in both 2D and 3D HEK-293 models. Notably, the cytoprotective effect in 3D tissues exceeded that of quercetin [40], indicating an enhanced effect in tissue-mimicking environments. Moreover, Elixspir^®^ markedly reduced intracellular ROS levels and increased cellular thiol content, pointing to the activation or preservation of intracellular redox systems, particularly glutathione-related pathways. The ability to modulate both extracellular radical scavenging and intracellular redox balance indicates a multi-targeted antioxidant mechanism, distinguishing Elixspir^®^ from conventional antioxidants that typically act through a single mechanism.

Given that UV-induced photodamage is largely mediated by ROS generation, these antioxidant findings prompted the hypothesis that Elixspir^®^ could also confer photoprotective benefits through similar redox-modulating mechanisms. Photodamage from UV radiation remains a primary contributor to extrinsic aging, with DNA fragmentation and ROS generation as key molecular events. Elixspir^®^’s photoprotective properties were confirmed through a combination of viability assays and single-cell gel electrophoresis (Comet assay). In particular, pre-treatment with Elixspir^®^ slightly enhanced post-irradiation cell viability, but more importantly, the Comet assay revealed a pronounced reduction in DNA fragmentation, suggesting protection at the genomic level. Therefore, our findings demonstrate, for the first time, that a light-colored aqueous Spirulina extract can provide effective protection against UV-induced DNA damage, representing a novel and unprecedented result in the field.

Compared to commonly used photoprotective ingredients such as ferulic acid or niacinamide, Elixspir^®^’s protective effect appears multifactorial, potentially involving both antioxidant-mediated ROS reduction and DNA repair modulation, though further molecular pathway analysis (e.g., Nrf2, p53) is needed to confirm this hypothesis.

Inflammation plays a central role in the aging process, as chronic low-grade inflammation, often referred to as “inflammaging”, contributes to the breakdown of skin structure, accelerates cellular senescence, and impairs tissue regeneration, ultimately leading to visible and functional signs of aging. The anti-inflammatory activity of Elixspir^®^ was evaluated by quantifying two well-characterized pro-inflammatory mediators, PGE2 and IL-1α in HEK-293 cells. Elixspir^®^ induced a significant 85.6% reduction in PGE2 levels, highlighting its ability to inhibit COX-mediated inflammatory pathways. Interestingly, IL-1α levels remained unchanged, suggesting a selective modulation of the inflammatory cascade. This selective inhibition profile could be advantageous, as it avoids full immunosuppression, maintaining some baseline immune vigilance while targeting key pathways involved in inflammation-induced skin aging. It is worth noting that, compared to well-known natural ingredients used for skin inflammation, such as licorice root or centella asiatica, which have shown broader anti-inflammatory activity [41,42], Elixspir^®^ provides a more focused modulation, potentially reducing off-target effects.

Finally, as hyperpigmentation is often a consequence of both chronic UV exposure and inflammation, making tyrosinase a prime molecular target [14], we thought it of interest to investigate whether Elixspir^®^ could modulate this pathway as part of its broader anti-aging potential. Elixspir^®^ demonstrated dose-dependent inhibition of tyrosinase, with 100% inhibition at 30% *v*/*v*, placing it on par with kojic acid, one of the most commonly used depigmenting agents. In addition, in the RHPE 3D tissue model, Elixspir^®^ significantly reduced melanin content, particularly at 25% w/w, with results comparable to kojic acid, but without evidence of tissue irritation. In contrast to kojic acid, which is associated with a risk of contact dermatitis and skin sensitivity with prolonged application, Elixspir^®^ demonstrates an excellent safety and tolerability profile. This distinction highlights its potential as a natural, non-irritating alternative for the treatment of pigmentation disorders.

## 4. Materials and Methods

### 4.1. Chemicals

The reagent tyrosinase (3490 U) was purchased from Sigma Aldrich (St. Louis, MO, USA). L-DOPA was purchased from Sigma Aldrich (St. Louis, MO, USA). Elixspir^®^, an 18% *w*/*w Arthrospira platensis* aqueous extract, was internally engineered according to an optimized procedure. Mass spectroscopy and specific colorimetric reactions were used to achieve a preliminary chemical characterization of hydrophilic constituents (see Supporting Information Table S1).

### 4.2. Cell Cultures

Human HEK-293 (ATCC CRL-1573) [43], HFF-1 (ATCC SCRC-1041) [44], and HaCaT [45] (Cell Line Service, DKFZ; Eppelheim, Germany) as well as Chinese hamster V-79 (ATCC CCL-93) [46] cell lines were cultured in 25 cm^2^ or 75 cm^2^ flasks and were maintained in logarithmic phase at 37 °C in a thermostatic incubator with a continuous flow of air, enriched with 5% carbon dioxide (CO_2_). The cells were grown in culture medium (DMEM or F-12 HAM’s, Euroclone, Milan, Italy) supplemented with 10% fetal bovine serum (Euroclone, Milan, Italy), antibiotics (penicillin: 50 units/mL; streptomycin: 50 μg/mL, Euroclone, Milan, Italy), and 2 mM glutamine (Euroclone, Milan, Italy).

### 4.3. Biocompatibility Studies

#### 4.3.1. MTT Assay

The cytotoxicity was evaluated using the MTT test as previously described [47]. Briefly, cells (3 × 10^3^ cells/well) are seeded in 96-well microplates in culture medium. After incubation at 37 °C with 5% CO_2_ for 24 h, the culture medium is removed and replaced with different concentrations of the extract. After an additional 24 h, each well was treated with a 5 mg/mL MTT saline solution, and after 5 h of incubation, 100 μL of isopropanol was added to each well. After an overnight incubation, cell growth inhibition was detected by measuring the absorbance of each well at 570 nm using a Bio-Rad 680 microplate reader (Bio-Rad, Hercules, CA, USA). The average absorbance of wells treated with different compound concentrations was expressed as a percentage of absorbance related to untreated control wells. Zinc diethyldithiocarbamate (ZDEC) and zinc dibutyldithiocarbamate (ZDBC) were used as reference standards in each plate. Evaluation was based on means from at least five independent experiments.

For phototoxicity studies, cells were seeded in 96-well microplates in culture medium; after incubation at 37 °C with 5% CO_2_ for 24 h, the culture medium was removed, and it was replaced with irradiated extract. Solutions with varying concentrations of Elixspir^®^ were initially irradiated for 15 min at 5 J/cm^2^ (UVA range) and then used for the treatment of the HEK-293 cell line as described above. Two UVA lamps (365 nm, Philips, Amsterdam, the Netherlands) were used as the irradiation source; prior to each experiment, both calibration and measurement of emitted energy (5 J/cm^2^) were performed using a radiometer.

#### 4.3.2. Spheroid Cultures and Acid Phosphatase (APH) Assay

Spheroid cultures were obtained by seeding 2.5 × 10^3^ cells/well in round-bottom suspension microplates (Greiner Bio-one, Kremsmünster, Austria) in phenol-red-free medium (Euroclone, Milan, Italy), which was supplemented with 20% methylcellulose to facilitate spheroid formation. An APH-modified assay was employed for evaluating cell viability in 3D spheroids, as previously described [47]. Briefly, the pre-seeded spheroids were treated with fresh medium containing the extract, and after 24 h, each well was treated with a 2 mg/mL solution of p-nitrophenyl phosphate (SigmaAldrich, Milan, Italy) in 0.1 M sodium acetate (pH 5.2) and 0.1% Triton X-100 and following 5 h of incubation, 10 μL of 1 M NaOH solution was added. The inhibition of cell growth induced by the tested complexes was detected by measuring the absorbance of each well at 405 nm using a Bio-Rad 680 microplate reader. Mean absorbance of the compound was expressed as a percentage absorbance related to untreated control wells. The average absorbance of wells treated with different compound concentrations was expressed as a percentage of absorbance related to untreated control wells.

### 4.4. Evaluation of the Photoprotective Effect

#### 4.4.1. Cell Viability

Experiments were performed following a previously reported protocol with only minor modifications [48]. HEK-293 cells (5 × 10^3^ cells per well) were seeded in 96-well microplates in culture medium. After incubation at 37 °C with 5% CO_2_ for 24 h, the culture medium was removed, and it was replaced with Elixspir^®^ at various concentrations. After one day of incubation, the microplates were irradiated for 30 min at 5 J/cm^2^ (UVA range), considering one control microplate without irradiation. Subsequently, after an additional 24 h, each well was treated with a saline solution of 5 mg·ml^−1^ MTT, and after another 5 h of incubation, 100 μL of isopropanol was added. The absorbance of each well at 570 nm was measured using a Bio-Rad 680 microplate reader. The average absorbance of wells treated with different extract concentrations is expressed as a percentage of absorbance relative to untreated control wells. The IC_50_ value represents the extract concentration that reduces the average absorbance at 570 nm by 50% compared to untreated control wells. The test was repeated on 5 independent cultures in triplicate. Two UVA lamps (365 nm, Philips) were used as the irradiation source. Prior to each experiment, both calibration and measurement of emitted energy (5 J/cm^2^) were performed using a radiometer.

#### 4.4.2. Comet Assay

Experiments were performed following a previously reported protocol with only minor modifications [49]. About 3 × 10^4^ HEK-293 cells were seeded in 25 cm^2^ flasks in a growth medium. After overnight incubation, cells were pre-treated for 6 h with Elixpir^®^ (5 mg/mL) and then irradiated for 30 min at 5 J/cm^2^ (UVA range). Cells were washed twice with cold PBS, harvested, and centrifuged, and DNA fragmentation was measured by the alkaline comet assay. Low-melting-point agarose, 300 μL (Trevigen, Inc., Gaithersburg, MD, USA) was heated to 37 °C and combined with 2 × 10^5^ cells/mL cell suspension. Each well of a 20-well CometSlide was filled with 50 μL of the cell/agarose suspension. The slides were placed in a 4 °C refrigerator in the dark for 15 min to solidify. Slides were then immersed in 50 mL of prechilled lysis solution (Trevigen, Inc., Gaithersburg, MD, USA). After draining excess liquid, the slides were transferred to 50 mL of freshly prepared (same day) alkaline DNA unwinding solution (200 mmol/L NaOH, 1 mmol/L EDTA, pH > 13) and incubated at room temperature in the dark for 20 min. After the unwinding step, electrophoresis was performed at 21 V for 30 min. Slides were then rinsed with distilled water and fixed for 5 min in 70% ethanol. Slides were dried and stained for 5 min at 4 °C with DAPI 5 μg/mL (Sigma-Aldrich, St. Louis, MO, USA). Pictures were captured with a Zeiss LSM-800 confocal microscope (Zeiss, Oberkochen, Germany). All photos were typeset in Zen 2.3 system (Zeiss, Oberkochen, Germany).

### 4.5. Skin Irritation Assay

Skin irritation was assessed using the SkinEthic™ Reconstructed Human Epidermis (RHE) model, a three-dimensional fully differentiated epidermal tissue composed of normal human keratinocytes cultured in a chemically defined medium at the air–liquid interface [33,34]. The SkinEthic™ RHE model was obtained from Episkin (Lyon, France); the reconstructed 0.5 cm^2^ epidermis was received on day 17 and maintained for 2 h in a SkinEthic growth culture medium (GCM) at 37 °C in 5% CO_2_.

The SkinEthic™ RHE model was used to evaluate skin irritation caused by Elixspir, following the Skin Irritation assay. This test follows OECD Test Guideline N° 439 for chemical testing. A 24-well plate was prepared, and the tissues were incubated at 37 °C with 5% CO_2_ for 2 h in a culture medium (SkinEthic™ GCM). Following this pre-incubation phase, the RHE tissues were topically exposed to 16 μL, equivalent to 32 μL/cm^2^, of Elixspir^®^ at concentrations of 5 mg/mL and 10 mg/mL for 42 min at room temperature. After exposure, the RHE tissues were rinsed 25 times with 1 mL of PBS. The treated tissues were then further incubated for 42 h at 37 °C with 2 mL of SkinEthic™ GCM. Tissues were exposed to phosphate-buffered saline (PBS) as a negative control.

After 42 h post-incubation at 37 °C with 5% CO_2_, tissue viability was assessed by the MTT reduction assay: RHE samples were transferred to a 24-well plate containing 200 μL of MTT solution (1 mg mL^−1^) and incubated for 3 h at 37 °C with 5% CO_2_. Formazan crystals were then extracted from the tissue by adding isopropanol (1.5 mL per well, for 2 h at RT). Tissue viability is reported as % of negative controls, measuring the absorbance of each isopropanol extract in triplicate at 570 nm by using a microplate reader (Bio-Rad 680 microplate reader). Results are the mean of three independent experiments.

Immediately after treatment (42 min exposure to Elixspir followed by 42 h of cell recovery), tissue lysate and culture media were collected and PGE2 and IL-1α were evaluated by specific ELISA assays (respectively, Enzo Life Sciences, Farmingdale, NY, USA, and Sigma-Aldrich) following the manufacturer’s instructions.

### 4.6. Antioxidant Activity

#### 4.6.1. DPPH Scavenging Activity

The assay was performed following previously reported materials and protocols, with minor modifications [50]. In the experiment, different volumes of Elixspir^®^, ranging from 0 to 2 mL were added to a cuvette, and the final volume was adjusted to 2 mL by adding ethanol. Subsequently, 1 mL of a 0.1 mM solution of 2,2-diphenyl-1-picrylhydrazyl (DPPH) was added to the cuvette. All samples were then placed in the dark for 15 min after shaking. After this incubation period, the absorbance of all samples was measured at 515 nm. The radical scavenging activity was expressed as the inhibition percentage and calculated using the following formula: (A_c_–A_s_)/A_c_∙100, where A_c_ is the absorbance of the control (absence of sample) and A_s_ is the absorbance of the sample. This formula quantifies the percentage of inhibition of the DPPH radical by the Elixspir^®^ extract.

#### 4.6.2. Evaluation of Cytoprotective Effect Against Oxidative Stress in 2D Cellular Models

The cytoprotective effect was evaluated by employing a previously reported method with minimal modifications [51]. Briefly, HEK-293 cells (3 × 10^3^ per well) were seeded in 96-well microplates in culture medium. After incubation at 37 °C with 5% CO_2_ for 24 h, the culture medium was removed, and Elixspir^®^ (0.01 mg/mL) or quercetin (50 µM) was added. After 24 h, oxidative stress was induced by adding H_2_O_2_ (250 µM, SigmaAldrich) for 6 h. Subsequently, each well was treated with 5 mg/mL MTT saline solution, and after 5 h of incubation, 100 μL of isopropanol was added to each well. After an overnight incubation, cell growth inhibition was detected by measuring the absorbance of each well at 570 nm using a Bio-Rad 680 microplate reader. The average absorbance of wells treated with different compound concentrations was expressed as a percentage of absorbance related to untreated control wells. Evaluation was based on means from at least five independent experiments.

#### 4.6.3. Evaluation of Cytoprotective Effects Against Oxidative Stress in 3D Cellular Models

The 3D cytoprotective effect was evaluated by adapting the previously reported method to spheroid cultures by means of the APH assay [47]. HEK-293 cells (2.5 × 10^3^ per well) were seeded by suspension in 96-well microplates in phenol-red-free medium (Euroclone, Milan), as previously described. After 72 h, the oxidative stress was induced by adding H_2_O_2_ (500 µm, SigmaAldrich) for 6 h. Subsequently, each well was treated with a 2 mg/mL solution of p-nitrophenyl phosphate (SigmaAldrich, Milan, Italy) in 0.1 M sodium acetate (pH 5.2) and 0.1% Triton X-100, and following 5 h of incubation, 10 μL of 1 M NaOH solution was added. Triplicate cultures were established for each treatment. The inhibition of cell growth induced by the tested complexes was detected by measuring the absorbance of each well at 405 nm using a Bio-Rad 680 microplate reader. Mean absorbance of the compound was expressed as a percentage absorbance related to untreated control wells. The average absorbance of wells treated with different compound concentrations was expressed as a percentage of absorbance related to untreated control wells.

#### 4.6.4. Evaluation of Cytoprotective Effects and Reduction in ROS Production in Response to Oxidative Stress

The production of ROS was measured in HEK-293 cells (10^4^ per well) grown for 24 h in a 96-well plate in phenol-red-free medium (Euroclone, Milan, Italy) as previously reported [47]. After 24 h, cells are pretreated with Elixspir^®^ (0.01 mg/mL) or quercetin (50 μM) for 4 h and exposed to oxidative stress induced by H_2_O_2_ (250 µM) for 2 h. Afterwards, cells were washed with PBS and loaded with 10 μM 5-(and-6)-chloromethyl-2′,7′-dichlorodihydrofluorescein diacetate acetyl ester (CM–H_2_DCFDA) (Molecular Probes-Invitrogen, Eugene, OR, USA) for 30 min in the dark. The increase in fluorescence over time is measured using a VICTOR Nivo Multimode Microplate Reader with excitation/emission wavelengths set at 495 nm and 530 nm, respectively; readings are recorded every minute for 1.5 h. Quercetin (25 µM) was used as a positive control.

#### 4.6.5. Determination of Redox State of Thiols

HEK-293 cells were seeded in 25 cm^2^ flasks and, after 24 h, were treated with the Elixspir (0.01 mg/mL) or quercetin (50 µM) for 24 h. Subsequently, the thiol content was measured as previously described [52]. Briefly, the day after the treatment, cells were washed with PBS buffer and centrifuged. Supernatants were removed, and cells were treated with 7 M guanidine in 0.2 M Tris-HCl (pH 7.5) containing 10 mM EDTA. Samples were subjected to two freezing/thawing cycles and treated with 3 mM DTNB to determine the content of total thiol groups using the Ellman assay. The change in absorbance was measured spectrophotometrically at 412 nm. As with cytoprotective effect assessment tests, quercetin (25 µm) was used as a positive control.

### 4.7. Anti-Inflammatory Activity

The quantitative determination of Prostaglandin E_2_ (PGE_2_) and Interleukin-1 alpha (IL-1α) was carried out using competitive immunological ELISA assays (PGE_2_-ELISA kit, Enzo Life Sciences; Human IL-1 α ELISA Kit, Sigma-Aldrich), following the manufacturer’s instructions [53,54]. Briefly, HEK-293 cells were seeded in 25-cm^2^ flasks and, after having reached 80% confluence, the cells were treated with the test substances at a concentration of 0.01 mg/mL, solubilized in phenol red-free medium. After 24 h of incubation, the cells were detached, centrifuged, and the supernatant was isolated. The concentrations of PGE_2_ and IL-1α were determined using a calibration curve.

### 4.8. Anti-Tyrosinase Activity

#### 4.8.1. Determination of the Tyrosinase Inhibition Activity of Elixspir

Tyrosinase activity was measured in phosphate buffer solutions (0.05 M, pH 6.8) by measuring the increase in absorbance at 475 nm as a result of the conversion of L-DOPA, a substrate, to DOPAquinone and DOPAchrome. An assay to determine the Elixspir^®^ tyrosinase inhibition was performed as described previously, with slight modifications [36]

To 2.5 mL of the solution of Elixspir^®^ extract at the desired % *v*/*v* (10–100% *v/v* in 50 mM PBS pH7) was added 10 µL of 10 U/mL of tyrosinase under stirring, and the resulting solution was left to incubate for 10 min at room temperature. Then 2.5 mL of 4 mM L-DOPA solution was added to reach the final desired concentration (5–50% of Elixspir^®^, 2 mM L-DOPA, and 2 U/mL tyrosinase), and the kinetic UV spectrum was recorded at 475 nm for 30 min to monitor the formation of DOPAquinone/DOPAchrome. To obtain the initial rate of the reaction (parameter used for comparing the enzyme activity), the slope of the straight line obtained by considering the first points of the intensity-time graph was calculated. The negative control was performed using 50 mM PBS pH 7 instead of Elixspir^®^ solution. Positive control was performed using 2.5 mL of 0.1% kojic acid.

#### 4.8.2. Depigmentation Assay

SkinEthic™ Reconstructed Human Pigmented Epidermis (RHPE) model (EPISKIN Laboratories, Lyon, France, EU) is reconstructed using cultured human keratinocytes combined with melanocytes of phototypes II, IV, or VI, situated in the basal layer [39]. For this test, tissues corresponding to phototype VI were utilized. Upon receiving the tissues, the RHPE inserts were transferred to a maintenance medium in a 6-well plate and incubated at 37 °C with 5% CO_2_ for 24 h in SkinEthic™ GCM culture medium. After this pre-incubation step, 10 μL (equivalent to 20 μL/cm^2^) of Elixspir solutions (25% *w*/*w* and 30% *w*/*w*) were applied daily for 4 consecutive days to the surface of the RHPE tissues in triplicate. Kojic acid (1% *w*/*w*), known for its whitening properties and ability to inhibit melanin production, was used as a positive control.

At the end of the treatment period, tissues (two per sample) were taken out of the inserts, and the melanin content was analyzed by adding 500 µL of 1 M NaOH containing 1% DMSO. Melanin extraction was carried out at 100 °C for 45 min, keeping the samples protected from light. The melanin concentration was determined by measuring absorbance at 490 nm using a microplate reader (Bio-Rad 680 microplate reader) and comparing the values to a calibration curve prepared with known concentrations of synthetic melanin. The results are reported as a percentage relative to the untreated control.

### 4.9. Statistical Analysis

All values are the means ± SD of no less than three measurements (starting from three different cell cultures in case of cell-based assays). Multiple comparisons were made by ANOVA followed by the Tukey–Kramer multiple comparison test, using GraphPad software version 10.4.2.

## 5. Conclusions

In conclusion, the positive outcomes from biocompatibility, cytotoxicity, and phototoxicity studies support the newly developed aqueous Spirulina extract Elixspir^®^ candidacy as a safe and well-tolerated well-aging component for skin formulations. In addition, being an aqueous Spirulina extract, it can be distinguished by the unique advantage of not imparting any color to cosmetic formulations, which is particularly beneficial for maintaining the esthetic appeal and versatility of skincare products. This characteristic greatly enhances consumer compliance by allowing formulators to create visually appealing, natural-like products without compromising on the efficacy or sensory experience. The efficacy and mechanistic studies revealed its intriguing potential not only as an antioxidant and anti-inflammatory active component but also as a brightening and photoprotective agent. However, further studies will be performed to deepen the understanding of the underlying molecular mechanisms and to validate the long-term benefits of Elixspir^®^ in diverse skin types and conditions.

Overall, our results revealed that the Spirulina extract Elixspir^®^ might represent a valuable alternative to classical synthetic components, especially for developing effective and safe natural-like anti-aging products, and laid down the basis for a broader application of Spirulina extract in the development of innovative skin care products.

## Figures and Tables

**Table 1:** Biocompatibility studies of Elixspir^®^ using MTT assay.

Cell-Line	IC_50_ (mg/mL)
HEK-293 (ATCC CRL-1573)	>10
V-79 (ATCC CCL-93)	>10
HFF-1 (ATCC SCRC-1041)	>10
HaCaT	>10

**Table 2:** Biocompatibility studies of Elixspir^®^ using APH assay.

Cell-Line	IC_50_ (mg/mL)
HEK-293 (ATCC CRL-1573)	>0.2
V-79 (ATCC CCL-93)	>0.2

## Data Availability

The original contributions presented in this study are included in the article/Supplementary Material. Further inquiries can be directed to the corresponding author.

## Supplementary Materials

The following supporting information can be downloaded at: https://www.mdpi.com/article/10.3390/ijms262311372/s1. References [55,56,57] are cited in the Supplementary Material.
