# Supplementary material for: Unveiling the Skin Anti-Aging Potential of the Novel Spirulina platensis Extract Elixspir®
Source: Int J Mol Sci. 2025 Nov 25;26(23):11372. doi: 10.3390/ijms262311372 (PMC12692491; doi:10.3390/ijms262311372)
Supplement: Supplementary file 1 [file ijms-26-11372-s001.zip › ijms-3879027-supplementary.pdf]

Supplementary material

Unveiling the Skin Anti-Aging Potential of the Novel *Spirulina platensis* Extract Elixspir®

Chiara Donati [1,†], Giulia Nerina Nardone [2,†], Vera Mason [2], Emanuela Di Gregorio [2], Irene Ragusa [2], Emanuele Amadio [2], Eleonora Zampieri [1], Rebecca Bassetto [2], Valentina Gandin [1,] and Samuele Zanatta [2,*]*

[1] *Dipartimento di Scienze del Farmaco, Via F. Marzolo 5, 35131 Padova, Italy*

[2] *Labomar S.p.a., Via F. Filzi 33, 31036 Treviso, Italy;*

[†] *These authors contributed equally to this work.*

* *Corresponding authors: Valentina Gandin, e-mail: valentina.gandin@unipd.it; Samuele Zanatta, e-mail: samuele.zanatta@labomar.com.*

Page(s)

Table of contents

Elixspir® preliminary chemical qualitative analysis (**Table S1**)	I-II
References	II

A preliminary chemical qualitative analysis was performed by High-Resolution Quadrupole Time of Flight (HR-QTOF) analysis. The Elixspir® 18% w/w aqueous extract was centrifuged before analysis. Table 1 summarizes the most abundant species detected using an untargeted QTOF-HR-ESI-MS approach.

Table S1. Qualitative HR-QTOF analysis of most abundant compounds detected.

m/z	Tentative identification	Adduct ion	Chemical Class
191.0195	Citric acid	$[M-H]^-$	Organic acid
238.2578	Tetrahydrobiopterin	$[M-H]^+$	Other
259.1297	gamma-Glutamyl(iso)leucine	$[M-H]^-$	Peptide
293.0676	Glutamylphenylalanine	$[M-H]^-$	Peptide
368.1206	Methoxycarbonylethyl-guanosine	$[M-H]^-$	Nucleotide
406.1258	Fructose malate fumarate	$[M-H]^-$	Carbohydrate
459.2156	Xyl(b1-2)Glc(b1-2)b-D-Fuc	$[M-H]^-$	Carbohydrate
493.1980<br>215.08	Fucose-PMP	$[M-H]^-$	Carbohydrate
509.1490	Hexose-PMP	$[M-H]^-$	Carbohydrate

215.08			

In addition, the total carbohydrate content was analyzed by the phenol–sulfuric acid method with glucose as a standard, and the protein content was determined using the Lowry assay by employing bovine serum albumin (BSA) as standard [S1][S2]. In addition, phycocyanin content was determined by the method of Boussiba and Richmond spectrophotometrically at 620 nm [S3].

These results confirmed that Elixspir® extract is characterized by a significant amount of carbohydrates (57.3 ± 4.6%) whereas phycocyanin and protein levels were very modest (6.0 ± 1.1% and 12.0 ± 3.2%, respectively).

References

[S1] Chen, W.; Gao, L.; Song, L.; Sommerfeld, M.; Hu, Q. An improved phenol-sulfuric acid method for the quantitative measurement of total carbohydrates in algal biomass. Algal Res. **2023**, 70, 102986.

[S2] Lowry, O.H.; Rosebrough, N.J.; Farr, A.L.; Randall, R.J. Protein measurement with folin phenol reagent. J. Biol. Chem. **1951**, 193, 265 − 275.

[S3] Boussiba, S.; Richmond, A.E. Isolation and characterization of phycocyanins from the blue-green alga Spirulina platensis. Arch Microbiol. **1979**, 120, 155–159.
